# Dementia risk prediction in early Parkinson's disease: Validation and genetic integration of the Montreal Parkinson risk of dementia scale (MoPaRDS)

**DOI:** 10.1177/1877718X251329857

**Published:** 2025-04-29

**Authors:** Aleksandra A Szwedo, Ingvild Dalen, Rachael A Lawson, Alison J Yarnall, Kenn Freddy Pedersen, Angus D Macleod, Carl E Counsell, David Bäckström, Lars Forsgren, Marta Camacho, Caroline H Williams-Gray, Ole-Bjørn Tysnes, Guido Alves, Jodi Maple-Grødem, On behalf of the Parkinson's Incidence Cohorts Collaboration

**Affiliations:** 1 The Centre for Movement Disorders, Stavanger University Hospital, Stavanger, Norway; 2 Department of Chemistry, Bioscience and Environmental Engineering, University of Stavanger, Stavanger, Norway; 3 Department of Research, Section of Biostatistics, Stavanger University Hospital, Stavanger, Norway; 4 Translational and Clinical Research Institute, Newcastle University, Newcastle upon Tyne, UK; 5 Department of Neurology, Stavanger University Hospital, Stavanger, Norway; 6 Institute of Applied Health Sciences, University of Aberdeen, Aberdeen, UK; 7 Department of Clinical Science, Neurosciences, Umeå University, Umeå, Sweden; 8 Department of Clinical Neurosciences, University of Cambridge, Cambridge, UK; 9 Department of Neurology, Haukeland University Hospital, University of Bergen, Bergen, Norway; 10 Department of Clinical Medicine, University of Bergen, Bergen, Norway

**Keywords:** Parkinson's disease, dementia, cognitive dysfunction, risk factors, genetic

## Abstract

**Background:**

Prediction models for dementia in Parkinson disease (PD) are needed to better identify high-risk patients, but existing risk models often lack validation in early-stage PD, when prognosis is most challenging.

**Objective:**

This study aims to validate the Montreal Parkinson Risk of Dementia Scale (MoPaRDS) in six population-based cohorts of newly diagnosed PD and to evaluate if incorporating genetic factors (*GBA1* and *APOE-ε4*) enhances its performance.

**Methods:**

We calculated MoPaRDS scores for 1108 newly diagnosed PD patients, and MoPaRDS + *GBA1 *+ *APOE* for the 941 patients with complete genetic data. We assessed the scores’ performance in predicting dementia diagnosed over 10 years using time-dependent receiver operating characteristic (ROC) curves.

**Results:**

Of the 1108 patients (mean age 69.5 ± 10.0 years; 61.0% men), 350 (31.6%) developed dementia. The area under the time-dependent ROC curve (AUC) was 0.79 for MoPaRDS and 0.80 for MoPaRDS + *GBA1 *+ *APOE.* Subdividing patients based on their MoPaRDS scores revealed annual observed risks of PDD of 39.4% (n = 8; high risk-), 11.4% (n = 176; intermediate risk-), and 5.0% (n = 942; low risk-group). With the suggested cutoff of ≥4, MoPaRDS had a sensitivity of 21.7% and specificity of 94.9%. Including the genetic items improved the sensitivity to 36.4% while maintaining comparable performance for specificity (91.5%).

**Conclusions:**

MoPaRDS demonstrates high specificity but limited sensitivity in early PD, highlighting that a one-size-fits-all approach is inadequate for predicting dementia risk in PD across different disease stages. Integrating genetic items increases sensitivity and identifies more newly diagnosed patients at higher risk of dementia, and may be a useful approach to assist dementia risk assessment in early-stage PD.

## Plain language summary

Many people with Parkinson's disease (PD) are at risk of developing dementia but predicting who will develop dementia early in the disease remains a challenge. Tools like the Montreal Parkinson Risk of Dementia Scale (MoPaRDS) aim to help identify patients at higher risk, but their accuracy in early-stage PD is not well established. This study tested the MoPaRDS tool in six large groups of newly diagnosed PD patients and explored whether adding genetic information (*GBA1* and *APOE*-ε4 genes) could make predictions more accurate. The study included 1108 people with newly diagnosed PD, of whom 350 (31.6%) developed dementia within 10 years. MoPaRDS was highly specific, meaning it could reliably identify people who were unlikely to develop dementia. However, it had limited sensitivity, identifying only 22% of those who later developed dementia. Adding genetic data improved sensitivity to 36%, meaning more at-risk individuals were flagged without losing significant accuracy. Further, the MoPaRDS tool can be used to group patients into low, intermediate, and high-risk categories. Patients in the high-risk group had a 39% annual chance of developing dementia, compared to only 5% in the low-risk group. Adding genetic information improved the tool's ability to identify more high-risk patients. These findings suggest that MoPaRDS is more appropriate for later-stage PD patients, and that incorporating genetic testing could improve existing tools for identifying those at higher risk of dementia, especially in the early stages of PD when intervention may be most beneficial. Such an approach could facilitate more tailored care for PD patients.

## Introduction

Dementia is a prevalent and serious complication in Parkinson's disease (PD), leading to poorer quality of life, increased need for institutionalized care, and a greater socioeconomic burden.^1,2^ A key strategy to enhance healthcare planning and effectively channel patients into clinical trials is the prediction of dementia risk in PD patients. Although various clinical, imaging, and biological factors have been linked to faster cognitive decline and PD dementia (PDD), the complexity of disease suggests that multivariable models are likely to provide a better prognostic accuracy than a single biomarker.^3^ Many of the models proposed to predict PDD require specialized tests or expertise that are not routinely available. Additionally, the majority were developed in either young, non-age-representative patients or prevalent PD.^4–9^ This lack of validation in diverse cohorts further restricts their applicability to the general PD population.

The Montreal Parkinson Risk of Dementia Scale (MoPaRDS) is a clinic-based screening tool designed to predict the risk of dementia in PD using eight standardized clinical and demographic items.^4^ The applicability of MoPaRDS may vary across different populations and clinical settings and there is limited data on the MoPaRDS performance in *de novo* PD. In this study, we aim to further validate MoPaRDS in an independent sample of 1108 newly diagnosed patients representative of the general PD population. We use data from up to 10 years of follow-up in six cohorts from the Parkinson's Incidence Cohorts Collaboration (PICC), a project pooling six population-based, prospective cohorts of newly diagnosed patients with PD. We also explore the value of adding two genetic biomarkers, *GBA1* and *APOE*-ε4, which have been previously associated with an increased risk of developing PDD in our study population.^10^

## Methods

### Study population

Patients in the study come from PICC, a collaboration between six population-based, Northern European cohorts of newly diagnosed PD recruited between 2000–2013: 140 patients from Cambridgeshire Incidence of Parkinson's disease from General Practitioner to Neurologist (CamPaIGN),^11^ 154 from Incidence of Cognitive Impairment in Cohorts with Longitudinal Evaluation-PD (ICICLE-PD),^12^ 144 from New Parkinson Patient in Umeå (NYPUM),^13^ 190 from Norwegian ParkWest (ParkWest),^14^ 279 from Parkinsonism: Incidence, Cognition and Non-motor heterogeneity in Cambridgeshire (PICNICS),^15^ and 201 from Parkinsonism Incidence in Northeast Scotland (PINE).^16^ Diagnosis was made according to UK Parkinson's Disease Society Brain Bank criteria without exclusion due to family history and all included patients had a confirmed PD diagnosis at the last clinical visit or postmortem. Patients were excluded from the PICC study if they were suspected of secondary parkinsonism (e.g., drug-induced or post-stroke parkinsonism) or other forms of parkinsonism, such as multiple system atrophy, progressive supranuclear palsy, corticobasal degeneration, or dementia with Lewy bodies. Other exclusions included monosymptomatic resting tremor, isolated gait disorder, isolated asymmetric tremor, essential tremor, or Alzheimer's disease. More details can be found in the publications for each cohort.^12–17^

### Ethical statement

Each study was approved by the relevant local review board: The Western Norway Regional Committee for Medical and Health Research Ethics (2010/1700), the Newcastle and North Tyneside Research Ethics Committee, the Regional Ethics Review Board in Umeå (03-387), the Multi-Centre Research Ethics Committee for Scotland, and the Cambridgeshire Local Research Ethics Committee (98/166). Written informed consent was obtained from all patients participating in the study (consent for research).

### Clinical assessments

Details of data collection in each cohort were described previously.^11–16^ Patients were systematically reassessed during visits at the clinic or, to reduce attrition, home visits or phone consultations. The assessment of parkinsonism was performed using Hoehn and Yahr staging^18^ and part III of the Unified Parkinson's Disease Rating Scale^19^ (UPDRS) in CamPaIGN, NYPUM, ParkWest, and PINE, or the Movement Disorders Society-UPDRS^20^ (MDS-UPDRS) in ICICLE and PICNICS. All scores are reported as MDS-UPDRS-III after conversion of UPDRS-III.^21^ Mini-Mental State Examination^22^ (MMSE) was used to assess global cognitive function.

### Risk scores

The MoPaRDS score was calculated as originally described by Dawson et al.^4^ If present at the baseline visit, a point was added for each of the eight items. The number of participants missing information is in brackets: Age over 70 years, Male sex, Falls and/or freezing (42), Bilateral disease onset (6), REM sleep behavior disorder (RBD) (436), Orthostatic hypotension (OH) (32), Mild cognitive impairment (MCI) (17), and Hallucinations (37). The assessments used to determine items are summarized in Supplemental Table 1. For the score including genetic items an additional point was added for both carriers of *GBA1* mutations (132) or *APOE*-ε4 (149), which were determined using a mix of whole exome sequencing, genotype arrays and probe-based genotyping assays, as described previously.^10^ As in the original study, values for missing variables were imputed as 0.5.

### Outcome assessment

We used clinical dementia diagnosis as the outcome in the study as described previously.^10^ Briefly, PDD diagnosis was made according to Diagnostic and Statistical Manual of Mental Disorders, 4th Edition (DSM-IV)^23^ and MMSE ≤24 in CamPaIGN, DSM-IV in PINE, or according to Movement Disorder Society criteria^24^ in ICICLE-PD, PICNICS, NYPUM, and ParkWest. As the time of onset of PDD, we used the midpoint between the last PDD-free visit and either the visit when PDD diagnosis was made or when PDD diagnosis was first recorded in clinical history or death certificate.

### Statistical analysis

Baseline demographic, clinical characteristics, and scores were summarized using descriptive statistics. Comparisons across cohorts were made using one-way analysis of variance (ANOVA), followed by Bonferroni post-hoc tests. Progression to PDD in the MoPaRDS risk groups was evaluated using Cox regression, with low risk as the reference group. Kaplan-Meier was used to visualize PDD-free survival and to estimate the mean time to PDD; as survival to PDD did not exceed 50% in the low-risk group, median times could not be calculated. Annual risk of PDD in each risk group was calculated as incidence rate percentage as follows: the number of PDD cases in the risk group was divided by the number of person-years at risk, multiplied by 100. The number of person-years was calculated based on death, loss to follow-up, or last recorded visit for those who did not develop PDD, and estimated time of PDD diagnosis for those who progressed to PDD. To evaluate the effect of each of MoPaRDS item on progression to PDD in our population, we performed Cox regression models stratified on study cohort. Patients were censored due to death, loss to follow-up, or last recorded visit. Survival analyses were performed using packages *survival* and *survminer*.

The discriminative ability of risk scores was evaluated using 10 years follow-up data in time-dependent receiver operating characteristic (ROC) curve analysis using the *timeROC* package.^25^ Time-dependent area under the ROC curve (AUC), cumulative sensitivity, dynamic specificity, positive predictive value (PPV), and negative predictive value (NPV) were estimated after the 9.5 years follow-up to avoid skewing of the estimates due to dense censoring for last available visit at 10 years. 95% confidence intervals (CI) were calculated based on the standard errors. Time-dependent AUCs were compared using the *compare* function in *timeROC* package.

Two-tailed p-values < 0.05 were considered significant and no adjustment for multiple testing was performed. Analyses were performed in IBM SPSS Statistics 29.0 (Armonk, NY, USA) and R 4.3.3.

## Results

### Study population and PDD prevalence

The study population, MoPaRDS items, and genetic data are summarized in Table 1. A total of 1108 patients were included in the pooled analysis, with 941 patients (84.9%) having data for both *GBA1* mutations or *APOE*-ε4 polymorphisms. The patients had a mean age at diagnosis of 69.3 ± 10.0 years and 61.0% were male. The median disease duration from diagnosis to baseline visit was 1.11 (interquartile range (IQR), 0–2.79) months. At the baseline visit, the average total MoPaRDS score was 2.45 (±1.31) and the average total MoPaRDS + *GBA1 *+ *APOE* score was 2.93 (±1.41). MoPaRDS scores differed significantly across the six cohorts (p < 0.001; Supplemental Table 2). Post-hoc comparisons revealed that ParkWest had lower MoPaRDS scores compared to CamPaIGN, ICICLE-PD, NYPUM, and PINE. Additionally, PICNICS had a lower score than NYPUM and PINE (all p < 0.05). The overall effect of cohort on MoPaRDS scores was small (η²p = 0.046), indicating that 4.6% of the variance in MoPaRDS scores was explained by cohort membership.

**Table 1.** Cohorts’ characteristics.

	Total PD	No PDD^a^	PDD^a^
N (%)	1108	758 (68.4)	350 (31.6)
Clinical variable, mean (SD)			
Age at symptom onset, y	67.5 (10.1)	65.9 (10.7)	70.8 (7.9)
Age at diagnosis, y	69.3 (10.0)	67.7 (10.6)	72.7 (7.8)
Age at baseline, y	69.5 (10.0)	68.0 (10.6)	73.0 (7.7)
Education, years	11.8 (3.5)	12.1 (3.5)	11.1 (3.4)
MDS-UPDRS III	31.3 (13.4)	29.7 (13.4)	34.7 (12.7)
Hoehn and Yahr, median (IQR)	2.0 (1.0)	2.0 (1.0)	2.0 (0.5)
MMSE, median (IQR)	29.0 (3.0)	29.0 (2.0)	28.0 (2.0)
Duration of follow-up, y			
Mean (SD)	6.2 (3.0)	5.9 (3.1)	6.9 (2.6)
Median (IQR)	6.3 (5.0)	6.0 (5.5)	7.2 (4.4)
Max	10.8	10.8	10.6
Deceased, N (%)	443 (40.0)	247 (32.6)	196 (56.0)
Lost to follow-up, N (%)	206 (18.6)	146 (19.3)	60 (17.1)
MoPaRDS variables, Item = 1, N (%)^b^			
Age ≥ 70 y	591 (53.3)	354 (46.7)	237 (67.7)
Male sex	676 (61.0)	453 (59.8)	223 (63.7)
Falls and/or freezing	378 (35.5)	229 (31.2)	149 (44.7)
Bilateral disease onset	238 (21.6)	146 (19.4)	92 (26.4)
REM sleep behavior disorder	120 (17.9)	62 (14.8)	58 (23.0)
Orthostatic hypotension	144 (13.4)	93 (12.6)	51 (15.2)
Mild cognitive impairment	219 (20.1)	99 (13.3)	120 (34.9)
Hallucinations	65 (6.1)	38 (5.2)	27 (8.1)
Total MoPaRDS score, mean (SD)^c^	2.45 (1.31)	2.22 (1.29)	2.95 (1.21)
Genetic variables, N (%) ^b^			
Any *GBA1* mutation	102 (10.5)	56 (8.4)	46 (15.0)
*APOE*-ε4	284 (29.6)	168 (25.6)	116 (38.2)
Total MoPaRDS + *GBA1 *+ *APOE* score, mean (SD)^c^	2.93 (1.41)	2.64 (1.37)	3.54 (1.32)

^a^ PDD diagnosed as described in Methods; No PDD group consisted of those who were not diagnosed with PDD before death, drop-out, or end-of-study.

^b^ Percentage calculated based on available data.

^c^ Scores calculated using 0.5 value for missing data.

Missing information: age at symptom onset (3), age at diagnosis (3), education (20), MDS-UPDRS III (15), MMSE (26); Falls and/or freezing (42), Bilateral disease onset (6), REM sleep behavior disorder (436), Orthostatic hypotension (32), Mild cognitive impairment (17), Hallucinations (37); Genetic variables: *GBA1* (132), *APOE-ε4* (149).

*APOE*: apolipoprotein E; CI: confidence interval; *GBA1*: glucocerebrosidase 1; IQR: Interquartile range; MDS-UPDRS: Movement Disorders Society Unified Parkinson Disease Rating Scale; MMSE: Mini-Mental State Examination; MoPaRDS: Montreal Parkinson Risk of Dementia Scale; N: number; PD: Parkinson's disease; PDD: Parkinson's disease dementia; REM: rapid eye movement; SD: standard deviation.

Among the enrolled patients, 350 (31.6%) developed dementia. The maximum observational period was 10.8 years, with a median follow up duration of 6.2 years (IQR 5.0). During the follow-up period, 443 (40.0%) patients died, and 153 (13.8%) patients withdrew for other reasons.

### Validation of the MoPaRDS score

**Figure 1.**
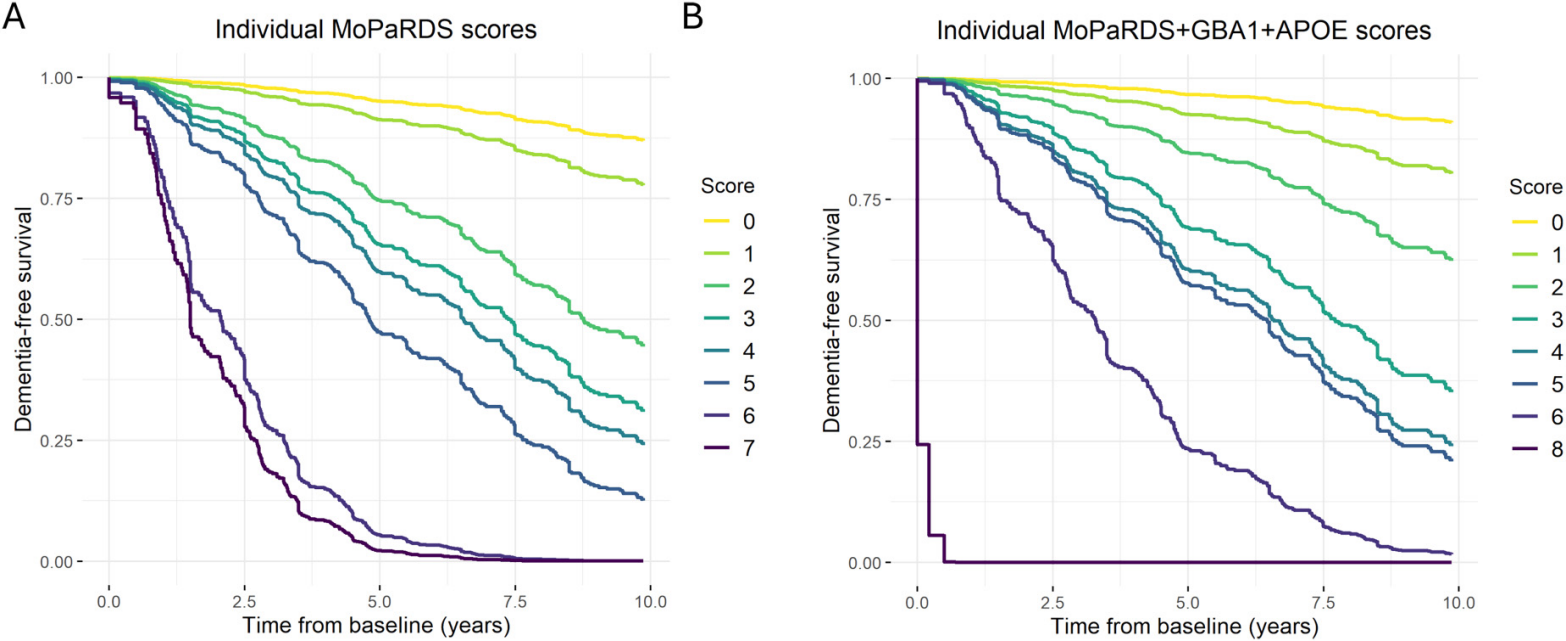
Survival analysis of dementia-free survival for the individual MoPaRDS and MoPaRDS + *GBA1 *+ *APOE* scores. The analyses were performed using unadjusted Cox regression models using the individual scores for the (A) MoPaRDS, including all 1108 patients, or (B) MoPaRDS + *GBA1 *+ *APOE, i*ncluding 941 patients with genetic data for both genes (no patients had a score of 7, 9 or 10). *APOE*: apolipoprotein E; *GBA1*: glucocerebrosidase 1; MoPaRDS: Montreal Parkinson Risk of Dementia Scale.

Over up to 10 years of follow-up, the MoPaRDS score demonstrated a robust predictive capability, with a time-dependent AUC of 0.79 (95% CI, 0.75–0.83). Excluding patients with missing data for any item (N = 498) did not markedly affect the AUC (0.78; 95% CI, 0.74–0.83). Increasing MoPaRDS scores significantly impacted the progression rate to PDD. Patients with higher scores had a sequentially increasing risk of progressing to PDD over the course of the study (Figure 1(a); Supplemental Table 3).

**Figure 2.**
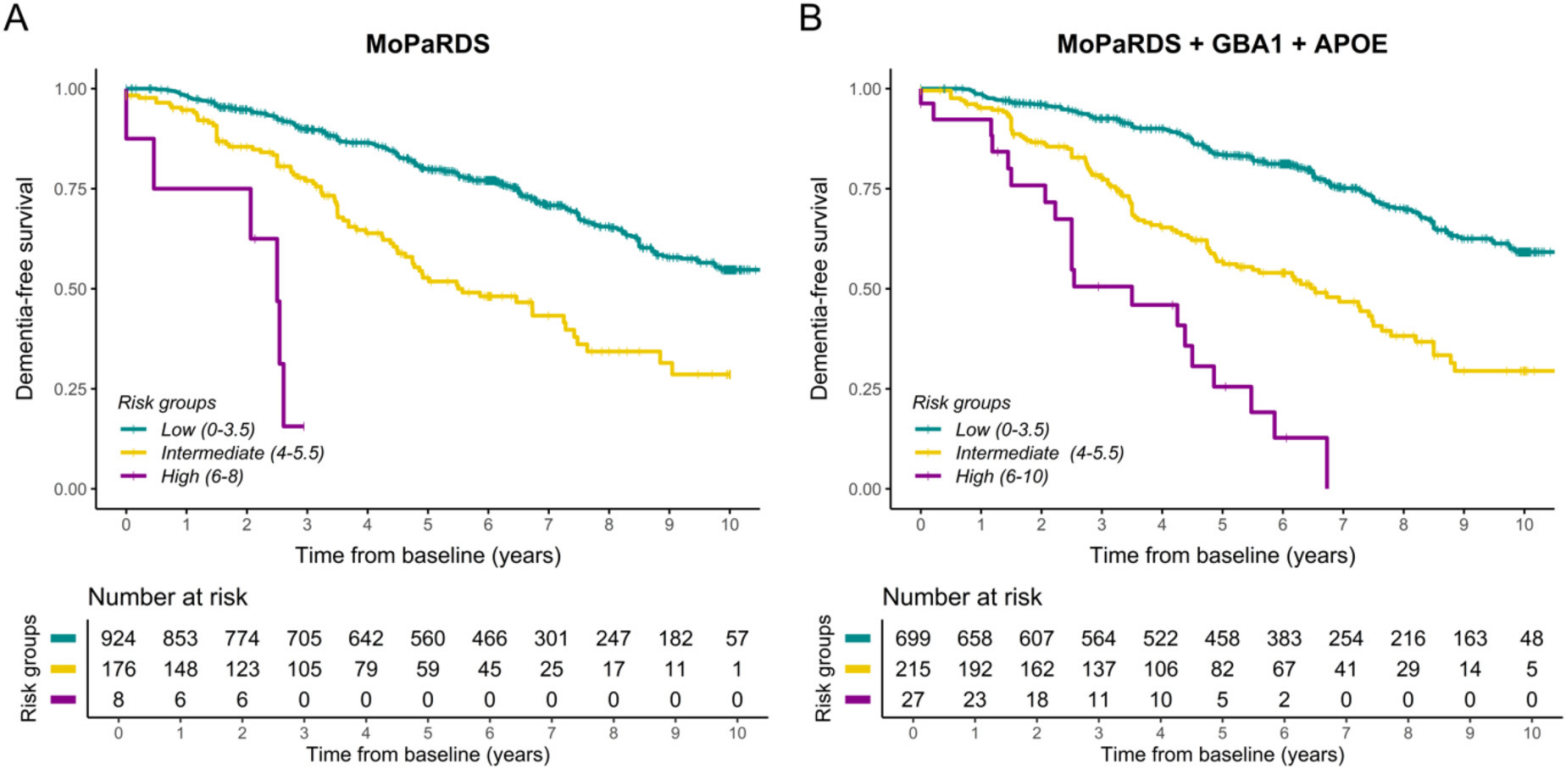
Progression to dementia by risk groups. Patients were grouped by risk group as outlined in the figure keys using either (A) the Montreal Parkinson Risk of Dementia Scale (MoPaRDS) in the total study population (N = 1108) or (B) MoPaRDS + *GBA1 *+ *APOE* in those with information available for both genes (N = 941). Censoring was by death, loss to follow up, or end-of-study as outlined in the methods. *APOE*: apolipoprotein E; *GBA1*: glucocerebrosidase 1; MoPaRDS: Montreal Parkinson Risk of Dementia Scale.

The MoPaRDS was first applied according to the original publication, categorizing the patients into low (0–3.5), intermediate (4–5.5), and high-risk (6–8) groups. The intermediate and high-risk groups had respectively 2.5 (95% CI, 2.0–3.3, *p *< 0.001) and 12.6 (95% CI, 5.5–28.8, *p *< 0.001) times higher risk of developing PDD compared to the low risk (reference) group (Table 2, Figure 2(a)). Most patients (924, 83.4%) scored in the low-risk group. In this group, 28.6% of patients eventually developed PDD, with an average time to dementia of 8.4 years (95% CI, 8.2–8.7 years) from diagnosis. The intermediate-risk group comprised 176 patients (15.9%), and 45.5% of these patients progressed to dementia. The average time to PDD for this group was shorter, at 6.0 years (95% CI, 5.4–6.5 years). The high-risk group included only 8 patients (0.7%) and notably 6 (75.0%) progressed to PDD within a short time (2.0 years, 95% CI, 1.2–2.7 years). Risk stratification revealed a clear gradient in the annual risk of developing PDD: the high-risk group had an annual risk of 39.4%, compared to 11.4% in the intermediate-risk group, and 5.0% in the low-risk group (Table 2).

**Table 2.** MoPaRDS score stratification into risk groups.

	PD/PDD (% PDD)^a^	Annual risk (%)	Mean time to PDD (y, 95% CI)^b^	HR (95% CI)	p
MoPaRDS					
Low risk (0–3.5)	924/264 (28.6%)	5.0	8.4 (8.2–8.7)	Ref.	
Intermediate risk (4–5.5)	176/80 (45.5%)	11.4	6.0 (5.4–6.5)	2.5 (2.0–3.3)	<0.001
High risk (6–8)	8/6 (75.0%)	39.4	2.0 (1.2–2.7)	12.6 (5.5–28.8)	<0.001
MoPaRDS + *GBA1 *+ *APOE*					
Low risk (0–3.5)	699/181(25.9%)	4.3	8.8 (8.5–9.0)	Ref.	
Intermediate risk (4–5.5)	215/100 (46.5%)	10.6	6.3 (5.8–6.9)	2.8 (2.2–3.6)	<0.001
High risk (6–10)	27/20 (74.1%)	25.4	3.5 (2.7–4.3)	8.0 (5.0–12.8)	<0.001

a The MoPaRDS was tested in the full population (N = 1108); MoPaRDS + *GBA1 *+ *APOE* in those with complete information on both genes (N = 941).

b Survival to PDD did not exceed 50% in the low-risk groups; therefore, median times could not be calculated, and mean times are reported.

*APOE*: apolipoprotein E; CI: confidence interval; *GBA1*: glucocerebrosidase 1; HR: hazard ratio; MoPaRDS: Montreal Parkinson Risk of Dementia Scale; PD: Parkinson's disease; PDD: Parkinson's disease dementia.

To further investigate the diagnostic performance of the MoPaRDS model in newly diagnosed patients, the intermediate and high-risk groups were combined. Applying the cutoff of ≥4, the score yielded a specificity of 94.9% (95% CI 91.6–98.1) but low sensitivity of 21.7% (95% CI 17.3–26.0). The PPV was high (79.4%; 95% CI 68.3–90.6) and the NPV was 57.1% (95% CI 52.6–61.6; Table 3). We then explored the performance of different cutoffs of MoPaRDS and observed that reducing the cutoff to ≥3 increased the sensitivity to 54.8% (95% CI 49.1–60.4) at the cost of a slight reduction in specificity (83.0%; 95% CI 77.4–88.5; Supplemental Table 4).

**Table 3.** Diagnostic evaluation of MoPaRDS models.

	All patients (N = 1108)	Patients with full genetic data (N = 941)	
Model	MoPaRDS	MoPaRDS	MoPaRDS + *GBA1 *+ *APOE*
Cutoff ≥ 4^a^			
Sensitivity, % (95% CI)	21.7 (17.3–26.0)	20.9 (16.3–25.5)	36.4 (30.8–42.1)
Specificity, % (95% CI)	94.9 (91.6–98.1)	95.2 (91.9–98.4)	91.5 (87.3–95.8)
PPV, % (95% CI)	79.4 (68.3–90.6)	79.1 (67.0–91.1)	79.0 (69.9–88.1)
NPV, % (95% CI)	57.1 (52.6–61.6)	57.8 (53.1–62.5)	62.1 (57.2–67.0)

^a^ Time-dependent cumulative sensitivity, dynamic specificity, PPV and NPV for MoPaRDS and MoPaRDS + *GBA1 *+ *APOE* scores over the 10-years follow-up time. 95% CI calculated based on the standard errors.

AUC: area under the ROC curve; *APOE*: apolipoprotein E; CI: confidence interval; *GBA1*: glucocerebrosidase 1; MoPaRDS: Montreal Parkinson Risk of Dementia Scale; N: number; NPV: negative predictive value; PPV: positive predictive value; ROC: receiver operating curve.

Finally, we evaluated the association of each MoPaRDS item individually and found that all items apart from male sex were associated with an increased risk of developing PDD (Supplemental Table 5; Supplemental Figure 1). However, in multivariate analysis including all items, only age over 70, RBD, MCI, and hallucinations reached statistical significance (p < 0.05; Supplemental Table 5).

### Value of genetic biomarkers with MoPaRDS for prediction in newly diagnosed PD

We tested the addition of carrier status for *GBA1* mutations and *APOE*-ε4 to the original MoPaRDS, which increased the total possible score to 10 points. The AUC of the original MoPaRDS (AUC 0.79, 95% CI 0.74–0.83) and the genetic MoPaRDS (AUC 0.80, 95% CI 0.75–0.84) were similar (*p *= 0.585) when compared in the 941 participants with complete genetic data. As with the clinical MoPaRDS, an increasing genetic MoPaRDS score was associated with a sequentially increasing risk of dementia (Figure 1(b); Supplemental Table 6).

Using the same risk group stratification as for the original MoPaRDS, both the intermediate (HR 2.8; 95% CI 2.2–3.6, *p *< 0.001) and high (HR 8.0, 95% CI, 5.0–12.8, p < 0.001) risk groups were found to have a higher risk of developing PDD compared to the reference group (Table 2, Figure 2(a)). After combining the intermediate and high-risk groups, at the cutoff of ≥4, the genetic MoPaRDS showed high specificity (91.5%; 95% CI 87.3–95.8) and nearly double the sensitivity (36.4%; 95% CI 30.8–42.1) compared to the MoPaRDS (Table 3). A similar pattern was observed at the lower cutoff of three, with high specificity in both scales (MoPaRDS: 83.0%, 95% CI 77.4–88.5 vs. genetic MoPaRDS: 73.9%, 95% CI 66.2–81.7), but higher sensitivity in the genetic scale (70.6%; 95% CI 64.8–76.3) compared to the clinical scale alone (54.8%; 95% CI 49.1–60.4), while retaining similarly high performance (>70%) across other parameters (Supplemental Table 6).

## Discussion

This study evaluates the performance of MoPaRDS, a clinic-based screening tool for estimating the risk of PDD, in population-based cohorts of patients newly diagnosed with PD. Using MoPaRDS stratification, we show a clear gradient in the risk of developing PDD, with the patients in the intermediate- and high-risk groups showing an increased likelihood of developing PDD within the first ten years after PD diagnosis. However, in its current form, MoPaRDS identified only a small number of higher risk individuals, reflecting that the burden of the clinical risk factors in the newly diagnosed PD population is low. Most patients (83%) were classified in the lowest risk group and exhibited an annual risk of developing PDD of 5.0%, a rate comparable to that observed the general PD population.^26^ We further show that the addition of genetic markers enhanced the scale's sensitivity and increased the number of individuals classified as intermediate or high risk. This improvement may allow for better detection of high-risk individuals before other clinical symptoms become evident.

### Validation of the original MoPaRDS

The original MoPaRDS was tested in a pooled group of three cohorts of patients with established PD (average disease duration ranging from approximately 3.8 to 7.1 years) and one *de novo* cohort (average disease duration 0.6 years) with an AUC of 0.879**1**. The references for this sentence need to be edited: References 2 and 28 are ok. Please remove 27 and 29. Add 30 (Anang et al 2017). Add New reference - The Parkinson Progression Marker Initiative. The Parkinson Progression Marker Initiative (PPMI). Prog Neurobiol 2011; 95: 629-35).^4,27–29^ However, the study populations lacked generalizability; many participants were originally involved in sleep studies, which may bias the composition of the cohort.^28^ Secondly, the *de novo* PPMI study participants were markedly younger at PD diagnosis than the general PD population.^30,31^ This, combined with the short follow up time, may account for lower incidence of PDD cases compared to the general *de novo* PD population, with only 13 (3.3%) converting to dementia after 4.4 ± 1.0 years of follow-up.^4^ By comparison, the dementia rate observed in population-based incidence PD cohorts after 4 to 5 years is between 17–25%.^32–34^

The MoPaRDS was later applied to a smaller subset of the PPMI cohort (N = 307) in a study which reported an unsatisfactory AUC of 0.66.^35^ Notably this study applied the scale to predict change in composite cognitive scores and not with a dementia diagnosis that the scale was developed for. Another study applied the MoPaRDS to a small (N = 48) independent population of prevalent PD with a minimum age of recruitment of 65 years (average 71.6 ± 4.8 years).^36^ In this population, MoPaRDS performed well (AUC 0.81), but only three items were independently associated with a dementia outcome (age, OH and MCI), which may reflect the small size of the cohort, or the features related to recruiting later stage patients who were dementia free after average disease duration of 8.9 ± 4.5 years.

Our study used an independent cohort of 1108 newly diagnosed PD cases that is age representative to the general PD population (average age of diagnosis 69.3 ± 10.0 years). Over the study period, 31.6% of the cohort developed PDD. The model had a good overall discriminative ability between positive and negative cases with an AUC of 0.79. However, using the recommended cutoff of ≥4, the scale displayed poor sensitivity (21.7%). Exploring different cutoffs, we found that reducing the threshold to 3 increased the sensitivity to 53.2% at the cost of a slight reduction in specificity. In comparison, the original study reported a higher sensitivity of 77.1% across pooled populations but did not specify the sensitivity for the *de novo* population.^4^ In the later study using a composite score for cognition and including only PPMI *de novo* patients, sensitivity was similarly low at 25%.^36^ These discrepancies indicate that the high sensitivity observed by Dawson et al. may be attributed to the patients with advanced PD being more likely to display a higher burden of risk signs, improving the model's predictive performance.

Of the individual MoPaRDS items, multivariate regression analysis showed that age over 70 and the presence of MCI, RBD or hallucinations significantly contributed to prediction, which aligns with well-established research and clinical observations.^37^ Notably, in the MoPaRDS, age is dichotomized into categories (<70 and ≥70), which offers simplicity but does not fully capture the gradual nature of increasing risk associated with age. Alternative approaches could consider categorizing age into more than two groups with different weightings or using age as a continuous variable to capture the full range of its influence on dementia risk.

### A composite MoPaRDS and genetic score

The MoPaRDS items were identified in a prevalent PD population^27^ and the underperformance of the MoPaRDS in the PICC cohorts compared to the original publication may be attributed to the differences in disease stage. In the early stages, the clinical predictors of dementia might be subtle or absent, making it challenging for the clinical score to detect those at risk. We and others have reported an increased risk of developing dementia in patients with PD who have inherited mutations in *GBA1* or carry the *APOE*-ε4 allele.^5,9,10^ This risk is evident at the time of PD diagnosis and integrating genetic information into a predictive scale could improve performance by informing on an individual's inherent susceptibility to developing dementia.

Previously, a large study of 3200 patients developed a clinical-genetic score for the prediction of cognitive decline in patients with PD.^9^ The authors reported the direct comparison of the clinical-only vs clinical-genetic score and found a small but significant improvement in AUC comparing the clinical (AUC 0.83) to clinical + *GBA1* (AUC 0.85) score. In our study, we found that the overall performance of the genetic MoPaRDS (AUC 0.80) was similar to the original MoPaRDs (AUC 0.79). However, the addition of the genetic items provided better sensitivity at all cutoffs compared to the clinical scale alone, while retaining comparable performance across other parameters. This demonstrates a practical improvement that might make the genetic MoPaRDS useful to support earlier and more personalized interventions based on specific risk profiles. For example, stratification into low, intermediate, and high-risk groups could provide a valuable framework for guiding patient care. In particular, as many of the MoPARDs items indicate high risk over short to medium term, patients in the high-risk group demonstrated a substantially elevated risk of progressing to PDD within a relatively short timeframe, warranting particularly vigilant follow-up and possibly earlier intervention strategies. Notably, work is needed to identify newly diagnosed patients at low risk of cognitive decline. Patients stratified into the lowest risk group had a comparable annual risk of PDD as the general PD population,^26^ while more promising with validation may be identifying patients scoring only 0 or 1 points. Identifying lower-risk individuals can help avoid unnecessary anxiety, reduce the burden of intensive monitoring, and allow healthcare resources to be more efficiently allocated.

### Strengths and limitations

One of the main strengths of this study is the use of deeply phenotyped, population-based cohorts of newly diagnosed patients, which made this the largest to-date validation study of the MoPaRDS in unselected patients. This is particularly important because population-based studies, unlike clinic-based studies which often recruit age-biased subjects, provide a more accurate representation of real-world PD populations.^31,32^ The MoPaRDS was applied to all patients shortly after diagnosis using accepted clinical scales and diagnostic criteria applied prospectively for up to ten years for both PD and PDD. Consequently, the PICC cohort can provide crucial information for understanding age-related outcomes such as dementia.

A potential limitation is the slight heterogeneity in the clinical assessments used to determine the MoPaRDS items across the cohorts. Further, two of the six cohorts (N = 419) did not include an assessment of RBD at the time of diagnosis and 167 participants did not have complete genetic data. Minor item variations and missing data are likely to appear across active clinics and represent the real-world utilization of such predictive scales. Further, while we found that MoPaRDS scores differed between some cohorts, the mean score differences between cohorts were <1 point for all comparisons, suggesting that, although statistical significance was achieved, the practical or clinical relevance of these differences is limited. As in the original study^4^ we did not exclude the patients with missing data and instead scored missing items 0.5 points, and this assumption may influence the predictive accuracy of the MoPaRDS. Notably, only 43 (3.9%) patients were missing data on two or more MoPaRDS items and sensitivity analysis including only participants with complete data revealed no notable difference in AUC. Regardless, future studies should aim to include comprehensive assessments to validate and potentially refine this scoring method.

To take advantage of the high-quality, extensive follow-up data we used time-dependent ROC analysis, which is better suited for analyzing longitudinal data than standard ROC analysis. However, due to the presence of a (semi)-competing risk scenario, treating death as a censored event in these and survival analyses may be a limitation.

## Conclusion

Our study highlights that a one-size-fits-all approach may not be ideal for predicting dementia risk in PD across different disease stages. The MoPaRDS, while demonstrating high specificity and positive predictive value, showed reduced sensitivity and negative predictive value in our cohort of newly diagnosed patients, suggesting it has limited utility as a standalone screening tool in early PD and may only be effective for flagging a very small number of patients at high risk of rapid progression to dementia. The integration of genetic factors (*GBA1* and *APOE-*ε4) enhanced the scale's sensitivity and increased the number of newly diagnosed patients identified as higher risk compared to the general PD population. This underscores the potential of incorporating genetic testing into risk assessment models to better tailor dementia prediction and screening strategies for early-stage PD.

## Supplemental Material

sj-docx-1-pkn-10.1177_1877718X251329857 - Supplemental material for Dementia risk prediction in early Parkinson's disease: Validation and genetic integration of the Montreal Parkinson risk of dementia scale (MoPaRDS) Supplemental material, sj-docx-1-pkn-10.1177_1877718X251329857 for Dementia risk prediction in early Parkinson's disease: Validation and genetic integration of the Montreal Parkinson risk of dementia scale (MoPaRDS) by Aleksandra A Szwedo, Ingvild Dalen, Rachael A Lawson, Alison J Yarnall, Kenn Freddy Pedersen, Angus D Macleod, Carl E Counsell, David Bäckström, Lars Forsgren, Marta Camacho, Caroline H Williams-Gray, Ole-Bjørn Tysnes, Guido Alves, Jodi Maple-Grødem and in Journal of Parkinson's Disease

## References

[1] Vossius C Larsen JP Janvin C , et al. The economic impact of cognitive impairment in Parkinson's disease. Mov Disord 2011; 26: 1541–1544. 21538519 10.1002/mds.23661

[2] Vatter S Stanmore E Clare L , et al. Care burden and mental ill health in spouses of people with Parkinson disease dementia and Lewy body dementia. J Geriatr Psychiatry Neurol 2020; 33: 3–14. 31146617 10.1177/0891988719853043

[3] Kalia LV . Biomarkers for cognitive dysfunction in Parkinson's disease. Parkinsonism Relat Disord 2018; 46(Suppl 1): S19–S23. 10.1016/j.parkreldis.2017.07.023 28781202

[4] Dawson BK Fereshtehnejad SM Anang JBM , et al. Office-based screening for dementia in Parkinson disease: the Montreal Parkinson risk of dementia scale in 4 longitudinal cohorts. JAMA Neurol 2018; 75: 704–710. 29582054 10.1001/jamaneurol.2018.0254 PMC5885166

[5] Phongpreecha T Cholerton B Mata IF , et al. Multivariate prediction of dementia in Parkinson's disease. NPJ Parkinsons Dis 2020; 6: 20. 32885039 10.1038/s41531-020-00121-2 PMC7447766

[6] Schrag A Siddiqui UF Anastasiou Z , et al. Clinical variables and biomarkers in prediction of cognitive impairment in patients with newly diagnosed Parkinson's disease: a cohort study. Lancet Neurol 2017; 16: 66–75. 27866858 10.1016/S1474-4422(16)30328-3 PMC5377592

[7] Ye BS Jeon S Ham JH , et al. Dementia-predicting cognitive risk score and its correlation with cortical thickness in Parkinson disease. Dement Geriatr Cogn Disord 2017; 44: 203–212. 28930751 10.1159/000479057

[8] Bäckström D Granåsen G Mo SJ , et al. Prediction and early biomarkers of cognitive decline in Parkinson disease and atypical parkinsonism: a population-based study. Brain Commun 2022; 4: fcac040. 10.1093/braincomms/fcac040 PMC8947320 35350553

[9] Liu G Locascio JJ Corvol JC , et al. Prediction of cognition in Parkinson's disease with a clinical-genetic score: a longitudinal analysis of nine cohorts. Lancet Neurol 2017; 16: 620–629. 28629879 10.1016/S1474-4422(17)30122-9 PMC5761650

[10] Szwedo AA Dalen I Pedersen KF , et al. GBA And APOE impact cognitive decline in Parkinson's disease: a 10-year population-based study. Mov Disord 2022; 37: 1016–1027. 35106798 10.1002/mds.28932 PMC9362732

[11] Williams-Gray CH Mason SL Evans JR , et al. The CamPaIGN study of Parkinson's disease: 10-year outlook in an incident population-based cohort. J Neurol Neurosurg Psychiatry 2013; 84: 1258–1264. 23781007 10.1136/jnnp-2013-305277

[12] Yarnall AJ Breen DP Duncan GW , et al. Characterizing mild cognitive impairment in incident Parkinson disease: the ICICLE-PD study. Neurology 2014; 82: 308–316. 24363137 10.1212/WNL.0000000000000066 PMC3929202

[13] Linder J Stenlund H Forsgren L . Incidence of Parkinson's disease and parkinsonism in northern Sweden: a population-based study. Mov Disord 2010; 25: 341–348. 20108376 10.1002/mds.22987

[14] Alves G Müller B Herlofson K , et al. Incidence of Parkinson's disease in Norway: the Norwegian ParkWest study. J Neurol Neurosurg Psychiatry 2009; 80: 851–857. 19246476 10.1136/jnnp.2008.168211

[15] Breen DP Evans JR Farrell K , et al. Determinants of delayed diagnosis in Parkinson's disease. J Neurol 2013; 260: 1978–1981. 23572347 10.1007/s00415-013-6905-3

[16] Caslake R Taylor K Scott N , et al. Age-, gender-, and socioeconomic status-specific incidence of Parkinson's disease and parkinsonism in northeast Scotland: the PINE study. Parkinsonism Relat Disord 2013; 19: 515–521. 23462482 10.1016/j.parkreldis.2013.01.014

[17] Foltynie T Brayne CE Robbins TW , et al. The cognitive ability of an incident cohort of Parkinson's patients in the UK. The CamPaIGN study. Brain 2004; 127: 550–560. 14691062 10.1093/brain/awh067

[18] Hoehn MM Yahr MD . Parkinsonism: onset, progression and mortality. Neurology 1967; 17: 427–442. 6067254 10.1212/wnl.17.5.427

[19] Fahn S Elton R and Members of the UPDRS Development Committee. Unified Parkinson's disease rating scale. In: S Fahn, CD Marsden, M Goldstein, DB Calne (eds.) Recent development in Parkinson’s disease. Florham Park: MacMillan Healthcare Information, 1987, pp.153–163.

[20] Goetz CG Tilley BC Shaftman SR , et al. Movement disorder society-sponsored revision of the unified Parkinson's disease rating scale (MDS-UPDRS): scale presentation and clinimetric testing results. Mov Disord 2008; 23: 2129–2170. 19025984 10.1002/mds.22340

[21] Goetz CG Stebbins GT Tilley BC . Calibration of unified Parkinson's disease rating scale scores to movement disorder society-unified Parkinson's disease rating scale scores. Mov Disord 2012; 27: 1239–1242. 22886777 10.1002/mds.25122

[22] Folstein MF Folstein SE McHugh PR . “Mini-mental state”. A practical method for grading the cognitive state of patients for the clinician. J Psychiatr Res 1975; 12: 189–198. 1202204 10.1016/0022-3956(75)90026-6

[23] American Psychiatric Association. Diagnostic and statistical manual of mental disorders: DSM-IV. Washington, DC: American Psychiatric Press Inc, 1994.

[24] Emre M Aarsland D Brown R , et al. Clinical diagnostic criteria for dementia associated with Parkinson's disease. Mov Disord 2007; 22: 1689–1707. quiz 1837. 17542011 10.1002/mds.21507

[25] Blanche P Dartigues J-F Jacqmin-Gadda H . Estimating and comparing time-dependent areas under receiver operating characteristic curves for censored event times with competing risks. Stat Med 2013; 32: 5381–5397. 24027076 10.1002/sim.5958

[26] Gibson LL Weintraub D Lemmen R , et al. Risk of dementia in Parkinson's disease: a systematic review and meta-analysis. Mov Disord 2024; 39: 1697–1709. 39036849 10.1002/mds.29918

[27] Anang JB Gagnon JF Bertrand JA , et al. Predictors of dementia in Parkinson disease: a prospective cohort study. Neurology 2014; 83: 1253–1260. 25171928 10.1212/WNL.0000000000000842 PMC4180482

[28] Anang JB Nomura T Romenets SR , et al. Dementia predictors in Parkinson disease: a validation study. J Parkinsons Dis 2017; 7: 159–162. 27911340 10.3233/JPD-160925

[29] The Parkinson Progression Marker Initiative. The Parkinson Progression Marker Initiative (PPMI). Prog Neurobiol 2011; 95: 629–635. 21930184 10.1016/j.pneurobio.2011.09.005 PMC9014725

[30] Beaulieu-Jones BK Frau F Bozzi S , et al. Disease progression strikingly differs in research and real-world Parkinson's populations. NPJ Parkinsons Dis 2024; 10: 58. 38480700 10.1038/s41531-024-00667-5 PMC10937726

[31] Macleod AD Henery R Nwajiugo PC , et al. Age-related selection bias in Parkinson's disease research: are we recruiting the right participants? Parkinsonism Relat Disord 2018; 55: 128–133. 29871791 10.1016/j.parkreldis.2018.05.027

[32] Williams-Gray CH Foltynie T Brayne CE , et al. Evolution of cognitive dysfunction in an incident Parkinson's disease cohort. Brain 2007; 130: 1787–1798. 17535834 10.1093/brain/awm111

[33] Pedersen KF Larsen JP Tysnes OB , et al. Natural course of mild cognitive impairment in Parkinson disease: a 5-year population-based study. Neurology 2017; 88: 767–774. 28108638 10.1212/WNL.0000000000003634

[34] Counsell C Giuntoli C Khan QI , et al. The incidence, baseline predictors, and outcomes of dementia in an incident cohort of Parkinson's disease and controls. J Neurol 2022; 269: 4288–4298. 35307754 10.1007/s00415-022-11058-2 PMC9294013

[35] Carlisle TC Medina LD Holden SK . Original research: initial development of a pragmatic tool to estimate cognitive decline risk focusing on potentially modifiable factors in Parkinson's disease. Front Neurosci 2023; 17: 1278817. 37942138 10.3389/fnins.2023.1278817 PMC10628974

[36] Bohn L McFall GP Gee M , et al. Dementia risk prediction in a longitudinal geriatric Parkinson's disease cohort: evaluation and application of the Montreal Parkinson risk of dementia scale. Can Geriatr J 2023; 26: 176–186. 36865405 10.5770/cgj.26.617 PMC9953498

[37] Aarsland D Kurz MW . The epidemiology of dementia associated with Parkinson's disease. Brain Pathol 2010; 20: 633–639. 20522088 10.1111/j.1750-3639.2009.00369.x PMC8094858

