# Supplementary material for: Dementia risk prediction in early Parkinson's disease: Validation and genetic integration of the Montreal Parkinson risk of dementia scale (MoPaRDS)
Source: J Parkinsons Dis. 2025 Apr 29:1877718X251329857. Online ahead of print. doi: 10.1177/1877718X251329857 (PMC13347466; doi:10.1177/1877718X251329857)
Supplement: Supplementary file 1 [file sj-docx-1-pkn-10.1177_1877718X251329857.docx]

# Supplemental Material

**Dementia risk prediction in early Parkinson’s disease: Validation and genetic integration of the Montreal Parkinson Risk of Dementia Scale (MoPaRDS)**

**Supplemental Table 1**. Assessments used to define items in MoPaRDS score in the PICC cohorts.

| **Item** | **Dawson et al.** | **CamPaIGN** | **ICICLE-PD** | **NYPUM** | **ParkWest** | **PICNICS** | **PINE** |
| --- | --- | --- | --- | --- | --- | --- | --- |
| **1. Age > 70** | Age older than 70 | Age at baseline ≤70 years (0); Age at baseline >70 years (1) | | | | | |
| **2. Male sex** | Male sex | Female sex (0); Male sex (1) | | | | | |
| **3. Falls and/or freezing** | MDS-UPDRS 2.12 scoring >1 and/or 2.13 scoring >0 | UPDRS-based: Item 2.13 = 0 and item 2.14 = 0 (0); Item 2.13 >0 or item 2.14 >0 (1) **OR**  MDS-UPDRS based: Item 2.12 ≤1 and item 2.13 = 0 (0); Item 2.12 >1 or item 2.13 >0 (1) | | | | | |
| **4. Bilateral disease onset** | Bilateral disease onset according to MDS clinical diagnostic criteria (subjective patient or caregiver report of bilateral onset and asymmetry index score <1.5 (higher MDS-UPDRS laterality scores (questions 3.3-3.8)/lower side MDS-UPDRS scores)) or a total difference of less than 3 across all MDS-UPDRS lateralized scores (sum of questions 3.3-3.8 on right vs left side) | UPDRS-based: Difference between the sum of right and left subscores in items 3.20 - 3.26 ≥3 (0); <3 (1) **OR**  MDS-UPDRS-based: Difference between the sum of right and left subscores in items 3.3 - 3.8 ≥3 (0); <3 (1) | | | | | |
| **5. RBD** | History suggestive of RBD that could be documented on clinical expert interview or with an RBD screening questionnaire score more than 5, or confirmed on polysomnogram according to standard criteria | Not available | MSQ RBD (Question 1: Do you act out dreams?), yes (1), no (0) | A question about history of disturbed dreams and violent movement, positive answer (1), negative answer (0) | Probable RBD: SSQ ^a^ score 2 or 3 (1) | Not available | A question about dream enacting behaviour, positive answer (1) |
| **6. OH** | Systolic blood pressure drop more than 10 mmHg standing compared with supine after 1 to 3 minutes | UPDRS item 4.42  0 (0), 1 (1) | BP measurements ^b^ | UPDRS item 4.42  0 (0), 1 (1) | BP measurements ^b^  **OR** UPDRS item 4.42  0 (0), 1 (1) | MDS-UPDRS item 1.12  0 (0), >0 (1) | BP measurements ^b^  **OR** UPDRS item 4.42  0 (0), 1 (1) |
| **7. MCI** | MDS Task Force PD-MCI guidelines or a MoCA score <26 | MMSE-based cutoff:  ≥26 (0), <26 (1) | Level 2 MDS criteria (1.5 SD below normative values) | Level 1 MDS criteria (1.5 SD below normative values) | Level 1 MDS criteria (1.5 SD below normative values) | MoCA/MMSE-based  both ≥26 (0), <26 (1) | MMSE-based  ≥26 (0), <26 (1) |
| **8. Visual hallucinations** | MDS-UPDRS 1.2 >0 | UPDRS item 1.2  <2 (0), ≥2 (1) | NMSQ item on hallucinations, yes (1), no (0) | UPDRS item 1.2  <2 (0), ≥2 (1) | UPDRS item 1.2  <2 (0), ≥2 (1) | MDS-UPDRS item 1.2  0 (0), >0 (1) | UPDRS item 1.2  <2 (0), ≥2 (1) |

^a^ SSQ: score 2 (very active during sleep, tend to wake up spouse); 3 (very active physically and verbally, have been hitting or hurting themselves or caregiver while sleeping)

^b^ Presence of OH defined as difference in supine and standing blood pressure measurements after active standing: systolic ≥ 20 or diastolic ≥10, or systolic ≥ 30 or diastolic ≥15 in those with supine hypertension defined as supine systolic ≥ 140 or diastolic ≥ 90. The period of active standing before measurements was 5 min in ICICLE-PD and 1 min in ParkWest and PINE.

BP: blood pressure; CamPaIGN: Cambridgeshire Incidence of Parkinson’s disease from General Practitioner to Neurologist; ICICLE-PD: Incidence of Cognitive Impairment in Cohorts with Longitudinal Evaluation-PD; MCI: mild cognitive impairment; MDS: Movement Disorder Society; MoCA: Montreal Cognitive Assessment; MoPaRDS: Montreal Parkinson Risk of Dementia Scale; MMSE: Mini-Mental State Examination; MSQ: Mayo Sleep Questionnaire; NMSQ: Non-Motor Symptoms Questionnaire NYPUM: New Parkinsonism in Umeå; OH: Orthostatic hypotension; PD: Parkinson’s disease; PICC: Parkinson’s Incidence Cohorts Collaboration; PICNICS: Parkinsonism: Incidence, Cognition and Non-motor heterogeneity in Cambridgeshire; PINE: Parkinsonism Incidence in North-East Scotland; RBD: rapid eye movement sleep behavior disorder; SD: standard deviation; SSQ: Stavanger Sleepiness Questionnaire; UPDRS: Unified Parkinson Disease Rating Scale.

**Supplemental Table 2**. Description of the six cohorts included in this study

|  | **CamPaIGN** | **ICICLE-PD** | **NYPUM** | **ParkWest** | **PICNICS** | **PINE** | **Total** |
| --- | --- | --- | --- | --- | --- | --- | --- |
| Patients included in the study, N | 140 | 154 | 144 | 190 | 279 | 201 | 1108 |
| Male, N (%) | 78 (55.7) | 100 (64.9) | 86 (59.7) | 115 (60.5) | 174 (62.4) | 123 (61.2) | 676 (61.0) |
| Age at baseline, y, mean (SD) | 70.6 (9.6) | 66.4 (10.4) | 71.2 (9.9) | 68.0 (9.3) | 68.7 (9.6) | 72.7 (10.3) | 69.0 (10.0) |
| Education, y, mean (SD) | 11.4 (3.7) | 12.8 (3.8) | 9.8 (3.4) | 11.1 (3.3) | 12.3 (3.2) | 12.4 (2.8) | 11.8 (3.5) |
| Total MoPaRDS score, mean (SD) | 2.47 (1.19) | 2.65 (1.50) | 2.79 (1.37) | 1.97 (1.28) | 2.30 (1.09) | 2.72 (1.35) | 2.45 (1.31) |
| Total MoPaRDS+*GBA1*+*APOE* score, mean (SD) | 2.92 (1.37) | 3.11 (1.59) | 3.23 (1.46) | 2.41 (1.35) | 2.74 (1.17) | 3.31 (1.47) | 2.93 (1.41) |

CamPaIGN: Cambridgeshire Incidence of Parkinson’s disease from General Practitioner to Neurologist; ICICLE-PD: Incidence of Cognitive Impairment in Cohorts with Longitudinal Evaluation-PD; MoPaRDS: Montreal Parkinson Risk of Dementia Scale; NYPUM: New Parkinsonism in Umeå; PICNICS: Parkinsonism: Incidence, Cognition and Non-motor heterogeneity in Cambridgeshire; PINE: Parkinsonism Incidence in North-East Scotland; SD: standard deviation.

**Supplemental Table 3**. Cox regression of the individual MoPaRDS scores.

|  | **MoPaRDS** | | | **MoPaRDS+*GBA1*+*APOE*** | | |
| --- | --- | --- | --- | --- | --- | --- |
| **Score range** | **PD/PDD, N (%)** | **HR (95% CI)** | **p** | **PD/PDD (%)** | **HR (95% CI)** | **p** |
| <1 | 96/8 (8.3%) | Ref. |  | 49/3 (6.1%) | Ref. |  |
| 1 - 1.5 | 257/37 (14.4%) | 1.8 (0.8 - 3.9) | 0.131 | 173/22 (12.7%) | 2.3 (0.7 - 7.7) | 0.178 |
| 2 - 2.5 | 299/104 (34.8%) | 5.8 (2.8 - 11.9) | <0.001 | 249/58 (23.3%) | 5.0 (1.6 - 15.8) | 0.007 |
| 3 - 3.5 | 272/115 (42.3%) | 8.4 (4.1 - 17.2) | <0.001 | 228/98 (43.0%) | 11.0 (3.5 - 34.6) | <0.001 |
| 4 - 4.5 | 122/53 (43.4%) | 10.2 (4.8 - 21.4) | <0.001 | 150/70 (46.7%) | 14.9 (4.7 - 47.5) | <0.001 |
| 5 - 5.5 | 54/27 (50.0%) | 14.8 (6.7 - 32.7) | <0.001 | 65/30 (46.2%) | 16.4 (5.0 - 53.9) | <0.001 |
| 6 - 6.5 | 6/5 (83.3%) | 57.4 (18.5 - 178.1) | <0.001 | 26/19 (73.1%) | 42.9 (12.6 - 146.0) | <0.001 |
| 7 - 7.5 | 2/1 (50.0%) | 74.5 (9.2 - 602.9) | <0.001 | 0 | NA | NA |
| 8 or 8-8.5 | 0 | NA | NA | 1/1 (100.0%) | 8603.0 (430.8 - 171802.1) | <0.001 |
| 9 - 9.5 | NA |  |  | 0 | NA | NA |
| 10 | NA |  |  | 0 | NA | NA |

HR estimated in an unadjusted Cox regression model. Due to missing data imputed as 0.5, fractional scores were combined with integers scores as indicated in the table. Analysis was performed in the 1108 patients (MoPaRDS) or 941 patients (MoPaRDS+*GBA1*+*APOE*).

*APOE*: apolipoprotein E; CI: confidence interval; *GBA1*: glucocerebrosidase 1; HR: hazard ratio; MoPaRDS: Montreal Parkinson Risk of Dementia Scale; N: number; NA: no value available; PD: Parkinson’s disease; PDD: Parkinson’s disease dementia.

**Supplemental Table 4**. Diagnostic evaluation of the MoPaRDS by cutoff.

| **Cutoff** | **PD-low risk/PD-high risk, N** | **Annual risk of PDD, %** | **Sensitivity,**  **% (95% CI)** | **Specificity,**  **% (95% CI)** | **PPV,**  **% (95% CI)** | **NPV,**  **% (95% CI)** |
| --- | --- | --- | --- | --- | --- | --- |
| ≥0 | 0/1108 | 5.9 | 100.0 (100.0 - 100.0) | 0.0 (0.0 - 0.0) | 47.7 (43.5 - 51.8) | NA |
| ≥1 | 96/1012 | 6.4 | 97.0 (94.8 - 99.2) | 15.9 (10.5 - 21.3) | 51.2 (46.8 - 55.7) | 85.4 (75.1 - 95.8) |
| ≥2 | 353/755 | 8.5 | 85.4 (81.1 - 89.6) | 57.4 (50.1 - 64.7) | 64.6 (59.1 - 70.1) | 81.1 (75.6 - 86.7) |
| ≥3 | 652/456 | 10.0 | 54.8 (49.1 - 60.4) | 83.0 (77.4 - 88.5) | 74.5 (67.3 - 81.7) | 66.8 (61.8 - 71.8) |
| ≥4 | 924/184 | 12.0 | 21.7 (17.3 - 26.0) | 94.9 (91.6 - 98.1) | 79.4 (68.3 - 90.6) | 57.1 (52.6 - 61.6) |
| ≥5 | 1046/62 | 15.8 | 7.9 (5.2 - 10.6) | 99.4 (98.3 - 100.0) | 92.7 (79.1 - 100.0) | 54.2 (50.0 - 58.4) |
| ≥6 | 1100/8 | 39.4 | 1.3 (0.3 - 2.3) | 100.0 (100.0 - 100.0) | 100.0 (100.0 - 100.0) | 52.6 (48.5 - 56.8) |
| ≥7 | 1106/2 | 46.9 | 0.2 (0.0 - 0.4) | 100.0 (100.0 - 100.0) | 100.0 (100.0 - 100.0) | 52.4 (48.2 - 56.5) |
| ≥8 | 1108/0 | NA | 0.0 (0.0 - 0.0) | 100.0 (100.0 - 100.0) | NA | 52.3 (48.2 - 56.5) |

Time-dependent parameters were calculated for all possible MoPaRDS cutoffs over the 10-years follow-up time. 95% CI calculated based on the standard errors. Analysis performed in the whole population of 1108 patients.

AUC: Area under the receiver operator characteristic curve; CI: confidence interval; MoPaRDS: Montreal Parkinson Risk of Dementia Scale; N: number; NPV: negative predictive value; NA: no value available; PD: Parkinson’s disease; PDD: Parkinson’s disease dementia; PPV: positive predictive value.

**Supplemental Table 5.** Cox regression analysis of MoPaRDS items and their association with progression to PDD in PICC cohorts over the 10 years follow-up.

|  | **PD = 1108, PDD = 350** ^a^ | | | **PD = 610, PDD = 225** ^b^ | |
| --- | --- | --- | --- | --- | --- |
| **Predictor variable** | **HR (95% CI)** | ***p*** | **HR (95% CI)** | | ***p*** |
| Age over 70 | 2.91 (2.31 - 3.66) | **<0.001** | 2.60 (1.94 - 3.48) | | **<0.001** |
| Male sex | 1.23 (0.99 - 1.53) | 0.067 | 1.17 (0.89 - 1.54) | | 0.273 |
| Falls or freezing | 1.61 (1.28 - 2.03) | **<0.001** | 1.27 (0.96 - 1.69) | | 0.095 |
| Bilateral onset | 1.68 (1.32 - 2.14) | **<0.001** | 1.03 (0.76 - 1.41) | | 0.848 |
| RBD | 1.84 (1.36 - 2.49) | **<0.001** | 1.90 (1.38 - 2.63) | | **<0.001** |
| OH | 1.42 (1.05 - 1.93) | **0.024** | 1.29 (0.90 - 1.85) | | 0.171 |
| MCI | 3.89 (3.04 - 5.00) | **<0.001** | 3.75 (2.79 - 5.04) | | **<0.001** |
| Hallucinations | 2.34 (1.52 - 3.62) | **<0.001** | 1.84 (1.10 - 3.09) | | **0.020** |

^a^ Univariate analysis including all participants (n = 1108). Each variable was evaluated in an individual Cox regression model stratified on the study cohort. Missing information: age at symptom onset (3), age at diagnosis (3), education (20), MDS-UPDRS III (15), MMSE (26); Falls and/or freezing (42), Bilateral disease onset (6), REM sleep behavior disorder (436), Orthostatic hypotension (32), Mild cognitive impairment (17), Hallucinations (37); Genetic variables: *GBA1* (132), *APOE*-ε4 (149).

^b^ Multivariate analysis including only individuals with no missing data (n = 610). All eight items were included in one multivariable Cox regression model stratified on the study cohort.

p values <0.05 are indicated in bold.

CI: confidence interval; HR: hazard ratio; MCI: mild cognitive impairment; MoPaRDS: Montreal Parkinson Risk of Dementia Scale; N: number; OH: orthostatic hypotension; PD: Parkinson’s disease; PDD: Parkinson’s disease dementia; PICC: Parkinson’s Incidence Cohorts Collaboration; RBD: rapid eye movement sleep behavior disorder.

**Supplemental Table 6.** Diagnostic evaluation of the MoPaRDS+*GBA1*+*APOE* by cutoff.

| **Cutoff** | **PD-low risk/PD-high risk, N** | **Annual risk of PDD, %** | **Sensitivity,**  **% (95% CI)** | **Specificity,**  **% (95% CI)** | **PPV,**  **% (95% CI)** | **NPV,**  **% (95% CI)** |
| --- | --- | --- | --- | --- | --- | --- |
| ≥0 | 0/941 | 5.7 | 100.0 (100.0 - 100.0) | 0.0 (0.0 - 0.0) | 46.8 (42.4 - 51.1) | NA |
| ≥1 | 49/892 | 6.1 | 98.7 (97.1 - 100.2) | 9.7 (5.1 - 14.3) | 49.0 (44.4 - 53.6) | 89.3 (77.0 - 100.0) |
| ≥2 | 222/719 | 7.4 | 90.6 (87.8 - 93.4) | 43.0 (35.4 - 50.7) | 58.3 (53.8 - 62.8) | 83.9 (77.4 - 90.4) |
| ≥3 | 471/470 | 9.8 | 70.6 (64.8 - 76.3) | 73.9 (66.2 - 81.7) | 70.4 (63.7 - 77.1) | 74.1 (68.6 - 79.6) |
| ≥4 | 699/242 | 11.7 | 36.4 (30.7 - 42.1) | 91.5 (86.2 - 96.8) | 79.0 (69.8 - 88.2) | 62.1 (57.1 - 67.1) |
| ≥5 | 849/92 | 14.2 | 14.4 (10.5 - 18.3) | 97.6 (95.2 - 99.9) | 83.9 (70.1 - 97.8) | 56.5 (51.8 - 61.2) |
| ≥6 | 914/27 | 25.4 | 5.5 (3.1 - 7.9) | 100.0 (99.5 - 100.0) | 100.0 (100.0 - 100.0) | 54.6 (49.2 - 60.1) |
| ≥7 | 940/1 | NA | 0.2 (0.0 - 0.5) | 100.0 (100.0 - 100.0) | 100.0 (100.0 - 100.0) | 53.3 (48.9 - 57.7) |
| ≥8 | 940/1 | NA | 0.2 (0.0 - 0.5) | 100.0 (100.0 - 100.0) | 100.0 (100.0 - 100.0) | 53.3 (48.9 - 57.7) |
| ≥9 | 941/0 | NA | 0.0 (0.0 - 0.0) | 100.0 (100.0 - 100.0) | NA | 53.2 (48.8 - 57.7) |
| >9 | 941/0 | NA | 0.0 (0.0 - 0.0) | 100.0 (100.0 - 100.0) | NA | 53.2 (48.8 - 57.7) |

Time-dependent cumulative sensitivity, dynamic specificity, PPV and NPV for MoPaRDS cutoffs over the 10-years follow-up time. 95% CI calculated based on the standard errors. Analysis performed in 941 patients with full genetic data available.

*APOE*: apolipoprotein E; AUC: area under the receiver operator characteristic curve; CI: confidence interval; *GBA1*: glucocerebrosidase 1; MoPaRDS: Montreal Parkinson Risk of Dementia Scale; N: number; NA: no value available; NPV: negative predictive value; PD: Parkinson’s disease; PPV: positive predictive value.

**Supplemental Figure 1**. Kaplan-Meier curves of dementia-free survival for Montreal Parkinson Risk of Dementia Scale items.

Dementia free survival was estimated for (A) Age over 70, (B) Male sex, (C) Falls and/or freezing, (D) Bilateral disease onset, (E) Rapid eye movement sleep behavior disorder (RBD), (F) Orthostatic hypotension (OH), (G) Mild cognitive impairment (MCI), (H) Hallucinations.
